# Robotic-assisted lobectomy after bilateral lung transplantation

**DOI:** 10.1093/icvts/ivac027

**Published:** 2022-02-03

**Authors:** Laura Romero-Vielva, Fernando Ascanio-Gosling, Anna González-Tallada, Joel Rosado-Rodríguez

**Affiliations:** 1 Thoracic Surgery and Lung Transplant Department, Vall d’Hebron University Hospital, Barcelona, Spain; 2 Anesthesia, Resuscitation and Pain Management Department, Vall d’Hebron University Hospital, Barcelona, Spain

**Keywords:** Robotic-assisted thoracic surgery lobectomy, Lung transplant, Non-small-cell lung cancer, Robotic-assisted thoracic surgery, Lung cancer

## Abstract

A 67-year-old woman diagnosed with a lung nodule on the left upper lobe was referred for surgical treatment. She had undergone a bilateral lung transplant in 2014. A robotic-assisted lobectomy with mediastinal lymph node dissection was performed. Final pathology revealed a pT3N0M0 Micropapillary Adenocarcinoma. The patient is alive and disease-free 13 months after the operation. This case report and video illustrate the safety and feasibility of a robotic-assisted lobectomy in a lung transplant recipient.

## INTRODUCTION

Minimally invasive techniques are the standard of care for the treatment of non-small-cell lung cancer. Robotic-assisted thoracic surgery (RATS) offers some advantages over video-assisted thoracic surgery as improved three-dimensional visualization and 7 degrees of freedom instruments. The RATS approach also offers advantages in cases of redo-surgery. *The disadvantages may be an increased surgical time and costs.*

Lung cancer is a major complication in recipients after lung transplant (LT) and should be managed according to the lung cancer treatment guidelines.

## CASE REPORT

**Figure 1:**
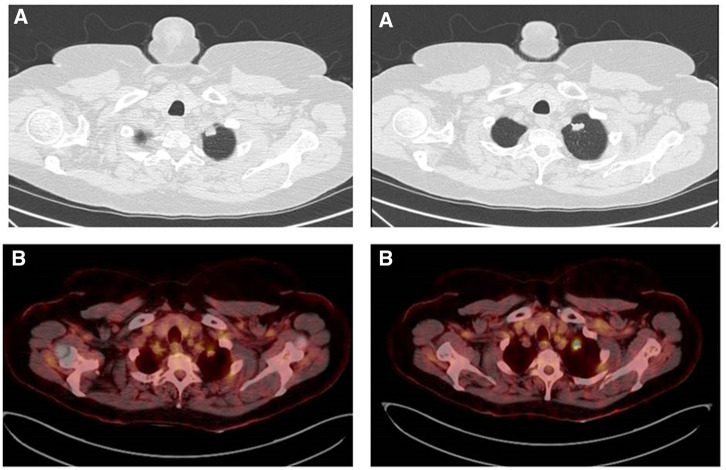
(**A**) Computed tomography showing a solid nodule in the left upper lobe. (**B**) Positron emission tomography showing a lung nodule with metabolic activity.

We present the case of a 67-year-old woman, diagnosed with a left upper lobe nodule. She had undergone a bilateral LT in 2014 due to chronic obstructive pulmonary disease. The donor was a never-smoker 54-year-old woman who died after a subarachnoidal haemorrhage. Sequential bilateral LT was performed through a Clamshell incision. The patient continued smoking 1 pack/day. An 8×15 mm subpleural nodule on the left upper lobe was seen on a computed tomography during the follow-up. The positron emission tomography showed an increased metabolic activity (SUVmax: 10.5; Fig. 1). Pulmonary function tests are shown in Table 1. The nodule was diagnosed with Adenocarcinoma (CK7 and TTF-1 positive) through fine-needle aspiration. The patient, ECOG grade 0, was receiving Tacrolimus, Mycophenolate Mofetil and steroids, the immunosuppressive protocol used in our LT programme. The case was discussed at a multidisciplinary board meeting, and an RATS lobectomy was planned. The patient was intubated with a single-lumen tube on the right bronchus and positioned on a right decubitus. We used the DaVinci Xi system with capnothorax. The camera and the 2 posterior ports were placed along the eighth intercostal space. The anterior port was placed in the seventh intercostal space. The assistant port was placed in a triangle between the anterior and the camera ports.

**Table 1:** Pulmonary function tests before and after RATS lobectomy

	17 March 2020		19 May 2021	
FVC	2.90 (l)	117.8%	2.10 (l)	83%
FEV1	2.12 (l)	109.6%	1.13 (l)	59%
DLCO	4.61 [mmol/(min*kPa)]	67.0%	2.63 [mmol/(min*kPa)]	39%
DLCO/VA	1.17 [mmol/(min*kPa*l)]	71.1%	0.90 [mmol/(min*kPa*l)]	55%

DLCO: diffusing capacity for carbon monoxide; FEV1: Forced expiratory volume; FVC: Forced vital capacity; DLCO/VA: diffusing capacity of the lung for carbon monoxide divided by alveolar volume.

The surgery started by liberating numerous adhesions, proceeded with a complete lymphadenectomy of levels 5, 7, 10 and 11, and lobectomy (video 1). The total surgical time was 393 min, and console time was 241 min, with an intraoperative blood loss of ∼300 ml. The drainage was removed on the eighth postoperative day due to prolonged air leak. Total length of stay was 10 days.

Pathology report showed a 1.7 cm Micropapillary Adenocarcinoma infiltrating the visceral and parietal pleura, pT3N0M0, no BRAF or EGFR mutations, and PDL-1, ALK, ROS1 negative. Adjuvant chemotherapy was not administered due to chronic kidney disease.

The pulmonary function test 11 months after surgery showed a decrease in FEV1 (Table 1). The patient is alive and disease-free 13 months after the operation.

## DISCUSSION

To our knowledge, this is the first case reported of an RATS lobectomy after a bilateral LT.

The feasibility and safety of RATS lobectomy have been demonstrated in the treatment of both early and locally advanced lung cancer and also offers advantages in cases of redo-surgery.[1–3].

Lung cancer is the third most frequent neoplasm in LT recipients with an incidence between 0.28% and 4.1%. COPD is considered a risk factor for lung cancer. The increased use of smoking donors may add a potential risk [4].

In this case, the donor was not a smoker, and the recipient had kept on smoking after the transplant; therefore, we assumed that smoking had increased the risk. The nodule, described as subpleural, was probably infiltrating the visceral pleura, therefore, the pT3 stage.

A successful case of video-assisted thoracic surgery lobectomy after a previous LT was reported by Decaluwe *et al.* [5]. Contrary to what they reported, we found increased adhesions in the whole pleural cavity. The robotic approach allowed us to take them down and a better visualization of the hilar structures. The three-dimensional vision and the wristed instruments could have made the dissection more precise and may have the potential for decreased the risk of bronchovascular injuries as suggested in previous reports [2].

## CONCLUSIONS

We present a successful RATS left upper lobectomy for a pT3N0M0 non-small-cell lung cancer in a patient with a previous LT.

RATS lobectomy may be feasible and safe to perform after previous thoracic surgery, including lung transplantation in selected cases.

**Conflict of interest:** Laura Romero-Vielva is a Host Surgeon for Abex Excelencia Robótica S.L. 

### Reviewer information

Interactive CardioVascular and Thoracic Surgery thanks Hiroshi Date and the other anonymous reviewers for their contribution to the peer review process of this article.
